# Single-Cell RNA Sequencing of Plant-Associated Bacterial Communities

**DOI:** 10.3389/fmicb.2019.02452

**Published:** 2019-10-29

**Authors:** Qin Ma, Heike Bücking, Jose L. Gonzalez Hernandez, Senthil Subramanian

**Affiliations:** ^1^Department of Agronomy, Horticulture, and Plant Science, South Dakota State University, Brookings, SD, United States; ^2^Biology and Microbiology Department, South Dakota State University, Brookings, SD, United States

**Keywords:** rhizosphere, microbiome, droplet-sequencing, split pool ligation-based transcriptome sequencing, fluorescence-activated cell sorting, rolling circle amplification, single primer isothermal amplification

## Abstract

Plants in soil are not solitary, hence continually interact with and obtain benefits from a community of microbes (“microbiome”). The meta-functional output from the microbiome results from complex interactions among the different community members with distinct taxonomic identities and metabolic capacities. Particularly, the bacterial communities of the root surface are spatially organized structures composed of root-attached biofilms and planktonic cells arranged in complex layers. With the distinct but coordinated roles among the different member cells, bacterial communities resemble properties of a multicellular organism. High throughput sequencing technologies have allowed rapid and large-scale analysis of taxonomic composition and metabolic capacities of bacterial communities. However, these methods are generally unable to reconstruct the assembly of these communities, or how the gene expression patterns in individual cells/species are coordinated within these communities. Single-cell transcriptomes of community members can identify how gene expression patterns vary among members of the community, including differences among different cells of the same species. This information can be used to classify cells based on functional gene expression patterns, and predict the spatial organization of the community. Here we discuss strategies for the isolation of single bacterial cells, mRNA enrichment, library construction, and analysis and interpretation of the resulting single-cell RNA-Seq datasets. Unraveling regulatory and metabolic processes at the single cell level is expected to yield an unprecedented discovery of mechanisms involved in bacterial recruitment, attachment, assembly, organization of the community, or in the specific interactions among the different members of these communities.

## Introduction

Plants are holobionts and are associated with complex and very diverse microbiomes (Simon et al., 2019). The long co-evolution of plants and their microbial communities has shaped the holobiont, and contributed to the development of microbial species that are specifically adapted to their respective plant host, and play a significant role in plant productivity and stress resistance. The microbiome or the second plant genome (Turner et al., 2013) represents a highly under-explored genetic resource with thousands of genes that can potentially be harnessed to increase crop yield and to alleviate stress responses. The advantages of using a microbiome-based solution include: (1) a typically shorter discovery to application pipeline due to a streamlined regulatory process, (2) a higher specificity compared to currently available crop protection products, and (3) a better compatibility with emerging precision agriculture technologies (Parnell et al., 2016; Deshayes et al., 2017). Due to the important role that the microbiome plays in plant health, stress resistance and nutrition acquisition, there is an increasing interest to design microbial communities that can promote plant growth in diverse environments. Plant-associated microbial communities are not randomly assembled; structure and composition of these microbiomes change in response to different environmental parameters (Bogino et al., 2013). Primary determinants of plant-associated microbial community composition and function include soil type (Lundberg et al., 2012), plant compartment (Bai et al., 2015), plant genotype (Bouffaud et al., 2014), activity of the plant immune system, and plant developmental stage (reviewed by Hassani et al., 2018). However, our current understanding about how microbial community compositions are shaped, how these communities are assembled, and how the interactions among specific bacteria affect the function of these communities is very limited.

Most plant-associated microbial communities, for example root surface bacterial communities, are spatially organized structures composed of root-attached biofilms and planktonic cells arranged in complex layers (Castiblanco and Sundin, 2016). In addition, evidence for bacterial co-association, symbiosis, and habitat sharing suggest that interactions among members might dynamically shape the community composition and function (Sloan and Lebeis, 2015). In this context, a microbiome can be compared to a multi-cellular organism, in which different cells serve distinct, but coordinated roles that control organismal function (Stovicek et al., 2012). The development of high throughput sequencing technologies allowed rapid and large-scale analysis of microbial ribosomal amplicons, metagenomes, or metatranscriptomes, and have provided us with insights into the taxonomic composition, collective gene pool, and gene expression patterns of microbiomes (Knief, 2014; Zhou et al., 2015). However, these methods are unable to reconstruct how gene pools and gene expression patterns are organized in individual cells of microbiomes. Single-cell genomics of microbial cells offers a solution to this limitation and can define the metabolic features and potential of individual cells that shape microbiome function. Application of single-cell approaches in microbes including single-cell transcriptomics of individual species have been reviewed recently (Chen Z. et al., 2017; Hwang et al., 2018; Zhang et al., 2018). Here, we discuss strategies on how currently available methods in single-cell RNA-Seq (scRNA-seq) including the highly scalable split pool ligation-based transcriptome sequencing (SPLiT-seq; Rosenberg et al., 2017, 2018) can be adapted for the exploration of plant-associated bacterial communities.

## Methods for Isolation of Single Bacterial Cells for Genomics Analyses

Methods for isolation of bacterial communities from plant root surfaces and apoplastic compartments without plant tissue contamination are available (White et al., 2015; McPherson et al., 2018; Gentzel et al., 2019). Isolation of bacterial cells from root surfaces typically involves sonication or vortexing in buffers containing mild detergents to dislodge cells from the root. Bacteria residing in plant apoplasts can be isolated by infiltrating the plant tissues with appropriate buffers followed by centrifugation to isolate the apoplastic wash fluids. Contamination with plant cells or other materials can be minimized by passing these fractions through a 20 μm sieve. These methods have been used to evaluate the composition of bacterial communities in a variety of plant species (Lundberg et al., 2012; Peiffer et al., 2013; Edwards et al., 2015; Wang et al., 2017; White et al., 2017). The same methods can be used to obtain source communities for single cell isolation and analysis. The compatibility of these methods with fixed cells negates any concerns on changes in microbial gene expression during the isolation process. Several approaches have been developed/adapted for genomics of single bacterial cells including serial dilution (Zhang et al., 2006), microfluidics (Chen et al., 2011), flow cytometry (Raghunathan et al., 2005), micromanipulation (Ishoy et al., 2006), or encapsulation in droplets (Tolonen and Xavier, 2017). The majority of these methods require the capability to isolate single cells and prepare individually labeled sequencing libraries from each of these cells. scRNA-Seq approaches of bacterial cells, however, are particularly challenging due to the lack of polyadenylated mRNAs and the lower number of template molecules per cell compared to those in plants and other eukaryotes. Strategies to adapt specialized methods to distinguish coding and non-coding RNAs and those for linear amplification of RNA/cDNA molecules for successful scRNA-Seq of bacterial cells are discussed in see section “Methods for construction of RNA-seq libraries from single bacterial cells”.

### Serial Dilution

A simple but effective method to isolate single cells from a bacterial population is serial dilution. After determining cell densities by direct counting via a Helber counting chamber or other reliable methods, cells are diluted to single cells into microtiter plate wells. These single cells can be enzymatically manipulated to lyse the cell wall and denature the membrane to release cellular contents for cDNA synthesis and library construction. Serial dilution has successfully been applied to single cells of *Escherichia coli* and *Procholorococcus* to develop a polymerase-based whole genome amplification method, polymerase cloning or “ploning” (Zhang et al., 2006). Serial dilution is an easy method that can be applied by most laboratories as it is simple and does not require any specialized instrumentation. One of the major limitations for this technique is, however, the risk of DNA contaminations from the environment or from reagents and labware that can lead to background amplifications. Strict sample handling and experimental protocols involving a clean air chamber and UV treatment of reagents and labware can lower these contamination risks. However, current assessments suggest that the precision of this methodology is insufficient, even if its accuracy of 88% is comparable to traditional flow cytometry-based technologies for single cell isolation (Raghunathan et al., 2005; Zhang et al., 2006).

### Micromanipulation

Many micromanipulation methods driven by the desire to culture single prokaryotic and eukaryotic cells were developed and improved throughout the last century (reviewed by Fröhlich and König, 2006). The low magnification of standard microscopical systems precluded their use for the isolation of single prokaryotic cells. Developments in resolution and magnification of modern microscopy has led to the adaptation of these methods for the investigation of larger prokaryotes such as filamentous bacteria (Kämpfer, 2006) and cyanobacteria (Šulčius et al., 2017). Micromanipulation has also been used to isolate individual bacterial cells from food samples (Hohnadel et al., 2018) and hot spring sediments (Ishoy et al., 2006). Two of the major approaches used in micromanipulation are (1) the use of a focused laser beam to capture and transfer the cell of interest from a population to a compartment (e.g., Keloth et al., 2018), and (2) the use of microinjectors in combination with the precision of a micromanipulator that can handle single prokaryotic cells (e.g., Ishoy et al., 2006). While the methodology is continuously improving and can be applied to address questions of organismal survival and success rate of recovery, it is laborious, very low throughput, and requires specialized equipment.

### Laser Capture Microdissection (LCM)

Laser Capture Microdissection is a contact- and contamination-free method for isolating specific single cells or entire areas of tissue from a wide variety of samples. In this technique the desired cell, or group of cells, is cut off a tissue section or other source, and is transferred without contact to a microtube for further processing (Nakazono, 2003). The advantage of this method is that it allows selecting individual cells of interest; but since the technique is very laborious and time-consuming, it only supports low throughput approaches. While this method has been used to for example study cell development in plant tissues or gene expression in mutualistic and pathogenic interactions (Balestrini et al., 2009; Gomez and Harrison, 2009), the insufficient spatial resolution makes this technique undesirable to isolate small bacterial cells from a dense community. Unlike eukaryotic cells that are in complex tissues, individual cells in bacterial communities can be easily separated by vortexing or other methods to obtain single cells. Therefore, other methods such as serial dilution (see section “Serial dilution”) or flow cytometry (see section “Fluorescence activated cell sorting”) may be more practicable than LCM. However, the ability to observe bacterial cells by LCM before they are selected provides some advantages, and the technique has been applied to isolate single bacterial cells from environmental samples. When plant microbe interactions are examined, LCM can be effectively applied to evaluate gene expression patterns in plant endophytes that are associated with specific regions of the plant. For example, root cortex and vascular tissues that are isolated by LCM can be subsequently used to evaluate single-cell genomics of endophytic microbes that reside within these tissues (Jahiri, 2013).

### Fluorescence Activated Cell Sorting

Fluorescence activated cell sorting (FACS) can be used to detect and sort cells from a population based on their different chemical or physical characteristics. Cells in suspension are transported, one cell at a time, and pass through a laser beam. Scattered light is characteristic of individual cells based on their composition and/or physical properties and is used to gate cells into collection chambers (Müller and Nebe-von-Caron, 2010). Typically, cells are labeled with one or more fluorescence markers to sort the cells into different chambers. This principle has been used to collect individual bacterial cells and determine their identities using multiple displacement amplification (Raghunathan et al., 2005).

One of the most common labels used for bacterial cell sorting is target-specific 16S rRNA fluorescence-*in situ*-hybridization (FISH). Limitations of the traditional 16S rRNA FISH technology, for example the limited and variable amounts of rRNA, have been addressed by the development of liquid phase tyramide signal amplification FISH (TSA-FISH), which is compatible with flow sorting. Similarly, custom made μFACS systems have been developed that support faster throughput and less expensive applications with lower contamination risks due to their use of closed systems, and their higher sorting efficiency (Chen et al., 2011). However, the sorting accuracy of these systems still needs to be significantly improved to be comparable to commercial cell sorters. However, single-cell genomics approaches do not rely on accurate sorting of different cell types, but rather accurate sorting of one cell per container (i.e., cell clumps must be avoided). Therefore, the combination of μFACS systems with viable cell deposition modules such as microwell arrays could make these technologies applicable despite their inaccuracies in sorting. The ability of μFACS systems to apply optical, electroosmotic, dielectrophoretic, and hydrodynamic switching methods for cell sorting offers advantages for their adaptability to a broad range of sample types (Müller and Nebe-von-Caron, 2010). Typical contamination risks from cell free DNA in liquid phase cell isolation systems particularly in environmental samples can be reduced in FACS/μFACS systems by multiple rounds of sorting (Chen et al., 2011).

### Droplet-Based Systems

Recent developments in microfluidic technologies have led to the development of instrumentation capable of sorting individual cells by encapsulating each of them in individually barcoded gel beads followed by the library preparation of individually barcoded RNAseq libraries. The current commercially available microfluidic platforms are the 10 X Genomics Chromium platform (Pleasanton, CA, United States) with a cell size range of up to 50 μm, and the Fluidigm C1 platform (South San Franscisco, CA, United States) with a cell size range of 10–17 μm. Recently, single-cell printing methods were adapted for the encapsulation of single bacterial cells in droplets (Riba et al., 2016). These systems use a transparent microfluidic drop-on-demand dispenser chip coupled with a camera-assisted automatic cell detection system. Cell detection and classification helps to avoid the collection of empty droplets and thus enables a “one cell per droplet” printing mode (Gross et al., 2013). Dispenser chips with smaller channel depth and nozzle to allow the detection and printing of cells down to 1 μm in size were developed for bacterial cells (Riba et al., 2016). However, in all these platforms, bacterial cell walls need first to be permeabilized in order to incorporate the barcoded beads. In recent studies, partial spheroplasts of yeast were generated by treating the cell suspensions with zymolyase to digest cell walls before the encapsulation (Gasch et al., 2017). A similar approach can be adapted for bacterial cells. The main advantage of microfluidics resides in the high throughput capabilities by which these systems can yield encapsulated cells; up to 100,000 or 800 cells in the 10 X and Fluidigm platforms, respectively.

Alternatively, it is possible to use custom-made drop-seq devices in combination with commercially available barcoded beads (Macosko et al., 2015). It is important to note that the approach also requires modifications in the cDNA synthesis phase due to the lack of polyadenylation of bacterial transcripts.

## Methods for Construction of RNA-Seq Libraries From Single Bacterial Cells

Many of the cell isolation methods have successfully been used to obtain genome sequences of individual bacterial cells, and can be adapted to obtain epigenomes as well. However, their adaptability for scRNA-seq is limited. Lack of polyadenylation in bacterial mRNAs, for example, requires methodologies to selectively enrich these molecules from the ∼10 times more abundant tRNA and rRNA molecules (see section “Methods to enrich bacterial mRNAs”). The very low RNA content of bacterial cells is another challenge, and requires the amplification of RNA or cDNA molecules, while amplification biases are avoided (see section “Methods to amplify RNA or cDNA”). Finally, the highly complex bacterial cell wall poses a challenge since enzymatic and chemical approaches to disrupt the cell wall and membrane may not be compatible with the reagents for the subsequent steps in RNA-seq library construction.

### Methods to Enrich Bacterial mRNAs

The need to prepare individually labeled sequencing libraries from individual cells precludes the use of typical affinity-based methods for enrichment of mRNAs or removal of rRNAs. In contrast, in-cell mRNA enrichment methods are suitable for this purpose.

#### Selective Exonuclease Based Enrichment of Messenger RNAs

Terminator^TM^ 5′-phosphate-dependent-5′- 3′exonuclease digests rRNAs and tRNAs, but not RNA molecules with a 5′-triphosphate, a 5′-cap, or a 5′-hydroxyl group. Consequently, Terminator^TM^ exonuclease can be used to selectively degrade rRNA and tRNA molecules with a 5′-phosphate structure, but not mRNA molecules. An exonuclease treatment at optimal concentrations enriched mRNA molecules from single cells of *Burkholderia thailandensis* and enables its potential use for next-generation sequencing (Kang et al., 2011, 2015). Measurements of different classes of RNA molecules by q-PCR indicated a significant reduction in the levels of tRNAs and rRNAs after exonuclease treatment. Comparison of gene expression profiles between enriched and unenriched samples using microarrays showed a negligible bias after the enrichment. While the use excess nuclease resulted in a better enrichment of mRNA molecules, a higher level of amplification bias was observed, possibly due to a non-specific digestion of mRNAs.

#### Reducing Non-desired Molecules in a Sequence-Specific Manner

There are two other methods with which rRNA or other non-target molecules can be reduced or eliminated. In the “not-so-random” (NSR) primer approach, random hexamers that match rRNA (or other non-target RNA) sequences are left out from the pool of random primers used for cDNA synthesis (Armour et al., 2009). This approach enriched non-rRNA derived cDNA molecules by four-fold (22% of the library to 87%). However, it is possible that a selected subset of hexamer primers can distort the resulting cDNA populations. In an alternate method, undesired sequences were eliminated after cDNA synthesis by random priming (Armour et al., 2018). Here, first strand synthesis was performed using random primers with an adapter containing a restriction enzyme recognition sequence. Subsequently, a second strand synthesis reaction was performed using primers specific to rRNAs or other undesired molecules resulting in double stranded cDNA molecules that contained these undesirable sequences. Next, restriction digestion was used to remove the adapters from these molecules and prevented their amplification during the next step of library preparation. In either case, prior knowledge about the undesirable sequences is required, what makes it difficult to use these methods for the evaluation of bacterial communities of unknown composition. A successful application of these methods for the enrichment of bacterial RNA has not yet been demonstrated.

### Methods to Amplify RNA or cDNA

#### Rolling Circle Amplification of RNA

Rolling circle amplification has been used to evaluate global gene expression in single cells of *B. thailandensis* (Kang et al., 2011). After cDNA synthesis, bacterial chromosomal DNA or other contaminant DNA molecules were digested by methylation-dependent restriction enzymes (e.g., McrBC and *Dpn*I), and the newly synthesized single-stranded cDNA (ss-cDNA) was circularized via 5′-end phosphorylation and intramolecular ligation. The circularized ss-cDNA was then randomly primed with RNA hexamers and subjected to multiple displacement amplifications using ϕ29 DNA polymerase. Thiophosphate-linked RNA random hexamers were used to reduce primer dimers and non-specific priming (Takahashi et al., 2009). This method yielded 25–30 μg cDNA from 0.2 to 1 pg RNA. The method was efficient and successfully amplified approximately 94–96% of the total transcripts. While absolute gene expression levels were poorly correlated between amplified single-cell transcriptomes vs. non-amplified bulk cell transcriptomes, the fold-change values were highly correlated. Using the same protocol single cell transcriptomes of *Pseudomonas aeruginosa* and *Burkholderia pseudomallei* were analyzed, and the authors suggested that the protocol might also be suitable for the analysis of mixed bacterial communities, and that identical amplification conditions for all samples should be used to avoid any interference from amplification bias (Kang et al., 2011, 2015).

### Single Primer Isothermal Amplification

In single primer isothermal amplification (SPIA), a unique chimeric 5′-RNA/DNA-3′ primer is used for first strand cDNA synthesis. Second strand synthesis results in a double-stranded cDNA with a unique DNA/RNA heteroduplex at one end. RNase H–mediated degradation of RNA in this heteroduplex enables binding of another chimeric 5′-RNA/DNA-3′ primer which is then extended to displace the existing first strand. The process of chimeric DNA/RNA primer binding, DNA replication, strand displacement and repeated RNA cleavage leads to a rapid accumulation of amplified cDNA (Kurn et al., 2005). SPIA was successfully used to obtain ∼7–17 μg of cDNA from just 5 fg of RNA from single cells of *Synechocystis* sp. PCC 6803. RNA-seq was used to evaluate changes in gene expression in three single cells each after nitrogen starvation. The average gene numbers in single cells were comparable to the numbers of bulk cell populations at each individual time point. Up to 98.6% of the genes in bulk cell populations were also detected in single cells underscoring the efficiency of SPIA amplification (Wang et al., 2015).

A number of other methods typically used for the amplification of polyadenylated mRNA molecules from single cells of eukaryotic organisms (Ziegenhain et al., 2017) can also be modified to amplify single-cell bacterial mRNA, but examples for the successful adaptation of these techniques are not yet available. Some of these techniques, such as single-cell universal poly(A)-independent RNA sequencing (SUPeR-seq) (Fan et al., 2015) are compatible with drop-seq and microfluidics-based approaches reviewed by Chen Z. et al. (2017). However, these methods might need to be preceded by mRNA enrichment as they do not distinguish between bacterial mRNAs, rRNAs or tRNAs.

## Adapting Split Pool Ligation-Based Transcriptome Sequencing for Bacterial Single-Cell RNA-Seq

**FIGURE 1:**
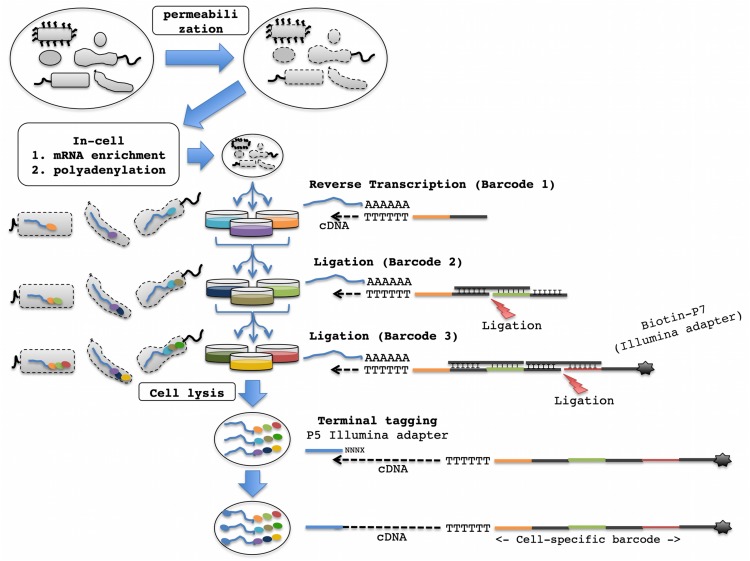
Proposed SPLiT-Seq workflow for scRNA-seq of bacterial communities. Following cell permeabilization and in-cell mRNA enrichment and polyadenylation, the cells go through three rounds of splitting and pooling, first for RT, followed by two rounds for adapter ligation. Wells of different colors depict distinct barcode sequences. Sequential addition of 3′ barcodes to a single mRNA molecule followed by terminal tagging to add the 5′ adapter is depicted on the right. The sequential addition of barcodes to mRNA molecules in different bacterial cells at each split-pool round is depicted on the left.

Split pool ligation-based transcriptome sequencing, a recently developed alternative for scRNA-seq, labels the cellular origin of RNA through combinatorial indexing (Rosenberg et al., 2018). This method has two major advantages: (1) it does not require the physical separation of single cells, and thus there is no need for specialized equipment; and (2) it uses unencapsulated and fixed cells and therefore provides maximized reagent compatibility with downstream molecular biological reactions for library construction (Rosenberg et al., 2017, 2018). For combinatorial indexing, (1) fixed and permeabilized cells are split into different microtiter plate wells, (2) a well-specific barcode is appended to intracellular transcripts, and (3) the cells are pooled back together (Figure 1). By repeating this process several times, each cell travels through a unique combination of wells with very high likelihood. Consequently, all transcripts from the same cell will contain a unique combination of barcodes indicating their cellular origin (Figure 1).

This method was originally developed for single-cell transcriptomics of mammalian cells, but could be ideal for scRNA-seq of bacterial communities due to its robust scalability in addition to the above described benefits. However, the presence of the bacterial cell wall and transcripts without a polyA tail will require the optimization of steps that are involved in cellular fixation, permeabilization, and in-cell library construction (see below). The successful application of methods for the permeabilization of bacteria for *in situ* hybridization and PCR as well as the selective enrichment and amplification of bacterial mRNAs are very promising for the effective adaptation of SPLiT-seq for bacterial scRNA-seq (Figure 1).

### Cell Permeabilization

Methods have already been established for the *in situ* localization of RNA through hybridization and PCR in a number of Gram-positive and Gram-negative bacteria (Tani et al., 1998; Russell and Keiler, 2009; Parsley et al., 2010). These methods can be used to effectively permeabilize bacterial cells for SPLiT-seq. Fixation of bacterial cells using para-formaldehyde and ethanol, followed by permeabilization using lysozyme and proteinase K treatment, for example, enabled the successful identification of specific transcripts in microbial communities through *in situ* reverse transcription (RT) and PCR with labeled nucleotides (Hodson et al., 1995; Tani et al., 1998). Since in-cell RNA-seq library construction essentially utilizes RT, adapter ligation, and PCR, we propose the use of these methods for the fixation and permeabilization of bacterial cells for SPLiT-seq.

### mRNA Enrichment

Terminator^TM^ 5′-phosphate-dependent-5′ – 3′exonuclease treatment to selectively enrich prokaryotic mRNA will be ideal for bacterial SPLiT-seq. Following mRNA enrichment, multiple options exist for amplification and library construction. For example, the remaining RNA molecules (primarily mRNAs) can be polyadenylated at the 3′-end using a Poly(A) polymerase in the presence of ATP (Figure 1). These molecules will now be compatible with the cell scRNA-seq library construction method that has been developed for eukaryotic cells (Rosenberg et al., 2017). However, the relatively small amounts of RNA in single bacterial cells might make efficient library construction difficult. In that case, the SPLiT-seq protocol can be easily modified to incorporate one of the RNA/cDNA amplification methods that have successfully been applied in prokaryotic systems (e.g., rolling circle amplification or SPIA, see above).

#### In-Cell Library Construction

The SPLiT-seq method involves in-cell RT using barcoded oligo-dT and/or random hexamer primers, followed by ligation of subsequent barcodes. Briefly, the cells are split into 48 wells each containing barcoded well-specific RT primers, and subjected to in cell RT reactions. If amplification is desired, random hexamer primers can be used in the RT step followed by barcoded primers for rolling circle amplification. This will be followed by two rounds of pooling and random splitting into 48 wells for barcode ligation (Figure 1). A total of three rounds of barcoding (one via RT and two via ligation) can sufficiently distinguish 100,000 single-cell libraries (48^3^ = 110,592 potential barcode combinations). The ligation plate wells would have dsDNA molecules with three distinct functional domains: a 5′-overhang that is complementary to the 5′-end on the cDNA molecule (originating from the RT primer), a unique well-specific barcode sequence, and the other 5′-overhang complementary to the 5′-overhang present on the DNA molecule that is ligated in the next ligation round (Figure 1). For the barcodes in the third round, the dsDNA molecules will have a 5′-overhang that is complementary to the 5′-end on the ligated cDNA molecule (originating from the previous round of ligation), a unique well-specific barcode sequence, and the other 5′-overhang with a universal PCR handle (suitable for Illumina Next-Gen sequencing and flow cell amplification), and a biotin molecule (Figure 1).

### Terminal Tagging and Amplification

After in-cell cDNA synthesis and a third round of barcoding, the cells can be lysed, and first strand cDNAs can be isolated by biotin-streptavidin affinity purification. A second Illumina compatible sequencing adapter will be appended to the 3′-end of the first strand cDNAs through terminal tagging (Illumina Script Seq kit; Figure 1). The resulting molecules that are tagged on both ends with Illumina-compatible adapters can be amplified, size-selected for 300–400 bp amplicons using solid phase reversible immobilization (SPRI) beads, and will be ready for next-generation sequencing. Based on the already available examples, we believe that adaptation of SPLiT-Seq for scRNA-Seq of bacterial communities will provide the entire plant-associated microbiome research community with a transformative technology to explore single-cell level changes in gene expression, and to spatially reconstruct microbiome processes.

## Bioinformatics of Bacterial ScRNA-Seq Data

### Read Mapping and Normalization

The major steps in the analytical pipelines for scRNA-seq typically mirror those used for bulk cell RNA-seq analysis. Reads from each cell after barcode splitting and quality control will be mapped separately to reference genomes. For a bacterial community RNA-seq, >13,500 complete bacterial genomes (NCBI as of 04/2019) is a good starting point, as a reference resource. Following regular read mapping (Langmead, 2010), normalization of read counts is a crucial step in RNA-seq analysis. It is generally agreed that compared to “within sample” normalization methods (e.g., FPKM - Fragments Per Kilobase per Million mapped reads), “between sample” normalization methods (e.g., TMM – Trimmed Mean of M values, DESeq) are more robust and accurate (Evans et al., 2018). However, the latter methods might perform poorly when zero counts are present due to a relatively large number of dropouts or cell-specific transcripts in scRNA-seq datasets. A recent method overcomes this by performing normalization based on summed expression values from pools of cells (Lun et al., 2016). While this improves normalization accuracy, it is obvious that the normalized expression values will only be applicable to the pools of cells, what makes this method undesirable for single-cell expression analysis. Therefore, the authors deconvolved the estimates for each pool into the estimates for its constituent cells, ensuring proper normalization of cell-specific biases. Therefore, pool-based normalized read counts can be effectively combined with differential expression analysis methods such as edgeR (McCarthy et al., 2012).

Due to the complex nature of microbiomes, one can expect a large number of unmapped reads. The use of single cell transcriptomes would allow generation and/or updating of reference resources. For example, *de novo* assembly of unmapped reads in each single cell to construct contigs using the assembly tool, Minia (Chikhi and Rizk, 2013) followed by scaffolding can be used to generate a new genome based on the Genome-organization-framework-assisted assembly pipeline and our previous knowledge of prokaryotic genome organization principles (Yin et al., 2010; Ma and Xu, 2013; Yuan et al., 2017).

### Bacterial Transcription Unit Profiling

Based on the normalized read counts data, the basic transcript units (TU) and their expression values are determined by counting the number of reads that map to each TU. Machine learning algorithms such as SeqTU also enable accurate prediction and identification of TUs (Chou et al., 2015). A web server of this algorithm^1^ was developed in 2017 and is available to automatically identify TUs with given RNA-seq data for any bacterium (Chen X. et al., 2017). In 2019, an R package was released to perform the TU identification locally (Niu et al., 2019). The predicted TUs are displayed intuitively using HTML format along with a graphic visualization of the prediction.

### Species and Cell Clustering

In scRNA-seq of bacterial communities, clustering based on their expression profiles and the cluster can be evaluated in two different ways: (1) biological process-based, and (2) taxonomy-based. In the first approach, each cluster is evaluated for enriched biological processes compared to other clusters using Gene set enrichment analysis (Subramanian et al., 2005) and the Database for annotation, visualization and integrated discovery (Dennis et al., 2003). This approach can be expected to determine distinct groups of cells within the microbiome that are enriched in distinct biological processes, for example chemotaxis, cell attachment, N fixation and metabolism, and cell multiplication. In addition, it may be possible to identify groups of cells with a distinct spatial location within the plant-associated microbiome based on their expression profiles; for example, those bacterial cells expressing attachment proteins are likely to be attached to plant surfaces, those expressing extra cellular matrix-associated proteins and displaying reduced expression of flagellar proteins are likely to be embedded in biofilms, and those expressing flagellar proteins are likely to be planktonic cells. In the second approach, the distribution of cells of the same species in different functional clusters and spatial groups can be evaluated. The results can be used to determine if and how different cells of the same species are functionally organized within the microbiome community at each given time point. Reconstructing the predicted microbiomes at each time point based on their spatial and functional information can be a crucial outcome of these analyses. A number of additional single-cell analytical tools are also available for these steps (see Table 1).

**TABLE 1:** Summary of popular analytical tools for scRNA-Seq.

**Tools**	**Year**	**Program**	**Tags**
SAMtools (Li et al., 2009)	2009	C	(Not scRNA-Seq specific) post-alignment processing
STAR (Dobin et al., 2013)	2013	C	(Not scRNA-Seq specific) alignment
Monocle2 (Qiu et al., 2017)	2017	R	Clustering, differential expression, dimensionality reduction, visualization
BackSPIN (Zeisel et al., 2015)	2015	Python	Gene filtering, biclustering, cell type prediction
SINCERA (Guo et al., 2015)	2015	R	Quality control, normalization, gene filtering, clustering, differential expression, marker genes, cell type prediction
MAST (Finak et al., 2015)	2015	R	Quality control, normalization, differential expression, network construction
Kallisto (Bray et al., 2016)	2016	C	Quantification
BPSC (Vu et al., 2016)	2016	R	beta-Poisson mixture model
salmon (Patro et al., 2017)	2017	C++	UMI, quantification
UMI-tools (Smith et al., 2017)	2017	Python	UMI, quantification
SC3 (Kiselev et al., 2017)	2017	R	Gene filtering, clustering, cell type prediction
Scater (McCarthy et al., 2017)	2017	R	Quantification, quality control, normalization, dimensional reduction, visualization
SCENIC (Aibar et al., 2017)	2017	R/Python	Clustering, network construction, regulon prediction, visualization
Seurat (Butler et al., 2018)	2018	R	Normalization, gene filtering, clustering, differential expression, marker gene, dimensionality reduction, visualization
SAVER (Huang et al., 2018)	2018	R	Imputation
SCDE (Fan et al., 2016)	2016	R	Differential expression, pathway analysis, visualization
GeneQC (McDermaid et al., 2018)	2018	Server	Alignment, mapping uncertainty, realignment, quantification
IRIS-EDA (Monier et al., 2019)	2019	Server Database	Correlation analysis, clustering, differential expression, visualization, dimensionality reduction
KEGG (Kanehisa et al., 2017)	2017		Gene annotation
EnrichR (Kuleshov et al., 2016)	2016	Database	Enrichment analysis
Harmonizome (Rouillard et al., 2016)	2016		Gene/protein function
SwissRegulon (Pachkov et al., 2012)	2013		Regulon database
reactome (Joshi-Tope et al., 2005)	2005		Gene annotation, pathway construction

*The tool names are hyperlinked to the relevant package, and the publication year is hyperlinked to the relevant reference.*

## Development of ScRNA-Seq Methods for Plant-Associated Bacterial Communities

### Establishment of Defined Microbial Communities for Method Development

We suggest selecting a defined microbial community with 8–10 distinct representative bacterial isolates. For example, to evaluate the assembly of a diazotroph community in cereal plant rhizospheres a mixture of *Herbaspirillum seropedicae (20%), Azospirillum brasiliense (5%), Bacillus thuringiensis (10%), Rhizobium leguminosarum (15%), Flavobacterium frigidarium (8%), Actinokineospora diospyrosa (12%), Bradyrhizobium* sp. *(20%)*, and *Methylibium* sp. (10%) can be used (Mao et al., 2014; Soni et al., 2017). A subset of ∼5,000 cells of this defined microbiome can be used to develop and optimize bacterial single-cell sequencing technologies. Single-cell transcriptomes of mammalian cells at a depth of 50,000 paired end reads per cell were sufficient to distinguish different stages of developing human neuronal cortex cells (Pollen et al., 2014). This and other similar studies showed that merged single-cell transcriptomes accurately represent a majority of the ensembled transcriptomes with strongly correlated expression levels. Plant-associated microbial communities on the other hand, contain uncharacterized species with genomes that are not as well annotated as the human genome. However, bacterial genomes typically have <5000 ORFs. Therefore, we expect that a sequencing depth of ∼100,000 reads per cell will allow a meaningful gene annotation and data interpretation.

### Evaluation of scRNA-Seq Results

Distinct benchmarks are essential to evaluate the results from scRNA-seq of the defined microbiomes and to validate the developed method for experimental samples. After reads are split according to barcode or assigned to individual microbial cells, they can be mapped to the known genomes of the 8 selected microbial species (see above) in a defined community. Ideally, all reads with the same barcode or those that came from a single cell should map to a single bacterial genome barring some highly conserved genes. Nevertheless, the results from these analyses can provide a benchmark to evaluate the accuracy with which scRNA-seq is able to distinguish transcripts from each individual bacterial species. Similar to the comparison of scRNA-seq data to bulk cell RNA-seq datasets from individual species (Kang et al., 2011; Wang et al., 2015), community scRNA-seq data need to be compared to metatranscriptomes of the same defined microbiome after mRNA enrichment and *in vitro* library construction. The results from this comparison can be used to evaluate the conformity of both data sets.

## Opportunities and Challenges in ScRNA-Seq of Plant-Associated Bacterial Communities

Evaluating the gene expression patterns in individual cells of plant-associated bacterial communities can provide transformative information not only about the gene expression levels and thereby function in individual members of different species but also about the spatial organization of bacterial communities in plant microbiomes. For example, cells with a higher expression of genes involved in exopolysaccharide synthesis are likely part of biofilms, while those expressing pili-encoding genes are likely attached to the plant surface. The estimated sequencing depth of 500 million reads per sample of 5000 cells [100,000 reads per cell] can be obtained from two sequencing runs (e.g., one high output run with ∼350–400 Mio reads and one medium output run with 120–130 Mio reads on an Illumina NextSeq500) making this approach relatively inexpensive given the depth of information obtained. One of the major challenges is the complexity associated with multiple bacterial genomes present in the community, and the extent of genome sequence information available for each species. Nevertheless, since transcripts from each cell are tagged, general functional capacities expressed in individual bacterial cells can be determined. In fact, it becomes increasingly clear that the metabolic functions of bacterial communities are more important than their taxonomic composition (Louca et al., 2016; Wallace et al., 2018).

## Author Contributions

SS conceptualized the review topic. All authors wrote and edited the manuscript.

## Conflict of Interest

The authors declare that the research was conducted in the absence of any commercial or financial relationships that could be construed as a potential conflict of interest.

## Footnotes

1 http://bmbl.sdstate.edu/SeqTU_dev/
